# The Relationship between Maladaptive Perfectionism and Anxiety in First-Year Undergraduate Students: A Moderated Mediation Model

**DOI:** 10.3390/bs14080628

**Published:** 2024-07-23

**Authors:** Zhiheng Xiong, Chunying Liu, Meila Song, Xiangzhen Ma

**Affiliations:** 1 School of Humanities, Southeast University, Nanjing 211189, China; xzh_psy@seu.edu.cn (Z.X.); 101012854@seu.edu.cn (M.S.); 2 School of Marxism, Nanjing Normal University of Special Education, Nanjing 210038, China; chunyingliu214@163.com

**Keywords:** first-year undergraduate students, maladaptive perfectionism, anxiety, mental health

## Abstract

To clarify the underlying mechanism of first-year undergraduate students’ maladaptive perfectionism in relation to their anxiety, this study constructs a mediating model with moderation, focusing on the investigation of the mediating role of self-compassion and the moderating role of family support. A total of 924 university students were involved in the investigation, responding to questionnaires on their maladaptive perfectionism, anxiety, self-compassion, and family support. The results showed that (1) after controlling for gender and Hukou, maladaptive perfectionism had a significant positive predictive effect on anxiety; (2) self-compassion can play a role in mediating the relationship between maladaptive perfectionism and anxiety; and (3) the mediating effect of self-compassion on anxiety in terms of maladaptive perfectionism was moderated by family support. The results of this study have important theoretical value and practical significance for improving first-year undergraduate students’ anxiety.

## 1. Introduction

First-year undergraduate students are in the transition from high school to university, with their learning styles and living environments undergoing dramatic changes [1]. During this process, students need to change their identities; leave their families, communities, and even their ethnic groups; complete an individual transformation; adjust certain habits formed during previous learning situations; and achieve a series of adjustments and changes between the two educational stages [2]. These complex issues often lead to increased pressure in many aspects of their lives, such as in their studies, finances, interpersonal communications, and life planning. Failure to address these difficulties adequately and adapt to the transition can lead to anxiety and a range of related symptoms, such as insomnia, loss of appetite, or more serious health problems [3]. Anxiety is a prominent negative emotion experienced by first-year undergraduate students. The anxiety detection rate was high in the results of this study, and the findings have important theoretical value and practical significance for improving first-year undergraduate students’ mental health, with about half of the students found to have anxiety symptoms, which is a gradual upward trend [4,5]. It has also been found that the detection rate of anxiety in first-year undergraduate students is higher than in other university students [6]. A series of research results show that a deep understanding of the factors affecting anxiety in first-year undergraduate students is significant for promoting an understanding of the current situation and preventing and reducing crisis events.

## 2. Literature Review

### 2.1. Maladaptive Perfectionism and Anxiety

Perfectionism is often characterised by the pursuit of perfection—setting too high a standard for oneself—accompanied by excessive strictness in self-evaluation and excessive attention to evaluation by others [7]. It is now widely accepted that perfectionism is a multidimensional construct, including both maladaptive and adaptive tendencies [8]. Among them, maladaptive perfectionists often set excessively high and unrealistic personal standards, struggle to feel satisfied even when they meet them, and believe that they can never do enough to be perfect [9]. They tend to blame their failures on their own shortcomings rather than external circumstances or factors [10]. This pursuit of perfection is usually accompanied by negative emotions, such as anxiety and depression [11]. To measure these two dimensions of perfectionism, Frost, Marten, Lahart, and Rosenblate (1990) developed the Frost Multidimensional Perfectionism Scale (F-MPS), which includes a number of aspects, namely, parental expectations (PE), parental criticism (PC), doubts about actions (DA), personal standards (PS), organisation (O), and concern over mistakes (CM) [7]. Subsequently, researchers empirically explored these six dimensions and summarised them into two core aspects: maladaptive evaluation concerns and positive striving [12]. Burgess further simplified the Frost Multidimensional Perfectionism Scale (F-MPS) by focusing on the personal standards (PS) and concern over mistakes (CM) dimensions, which are categorised as evaluative concerns (EC) and striving (S) [13]. EC reflect the tendency to be self-critical and overly concerned about negative evaluations due to mistakes or failures, while S reflects high goal attainment and personal standards [14]. However, several studies in the Chinese cultural context have viewed personal standards as being connected to maladaptive perfectionism [15,16,17]. This may be because Chinese culture emphasises collectivism and social acceptance, which often gives personal standards a more unique meaning [18,19]. When individuals pursue high standards accompanied by excessive self-criticism and worry, it can lead to depression, anxiety, and a fear of failure [20,21,22,23].

Anxiety generally refers to negative physiological, emotional, and behavioural responses, such as tension, discomfort, and worry, which individuals experience in response to potentially threatening situations [11]. As a psychological condition closely linked to environmental and personal stressors, anxiety is increasingly prevalent among college students and has become a significant threat to their lives and studies [24]. Maladaptive perfectionism is considered a risk factor for anxiety, with numerous studies demonstrating an association between maladaptive perfectionism and anxiety [25,26]. Maladaptive perfectionists tend to strive for excellence, setting themselves very high standards, with the hope of achieving the best results and performance in all aspects of their lives [27]. They may spend a lot of time and energy on their studies, pursuing perfect grades and recognition [28]. However, as their academic burden increases, they may feel that there is not enough time and that they are not able to fully complete the tasks or meet their expectations, and, thus, these high personal standards can cause anxiety and tension [29]. In addition, maladaptive perfectionists are often afraid of failure because they consider any imperfect outcome to be a failure [30]. This mentality may lead to the avoidance of challenges and the reluctance to take on tasks that they are less confident about, thus limiting their growth and development. Therefore, maladaptive perfectionism may intensify anxiety. Based on the above discussion, this study proposes Hypothesis 1: maladaptive perfectionism significantly predicts first-year undergraduate students’ anxiety.

### 2.2. The Mediating Role of Self-Compassion

Self-compassion refers to an individual’s ability to maintain sympathy in regard to the self in the midst of difficulties and pain. It is a high-value self-schema-related structure, which is particularly important for preventing the consequences of difficulties and setbacks [31] through recognition and a positive emotional response [32]. The undergraduate stage is a challenging and stressful time during which university students are faced with a series of complicated problems such as increased academic demands, social pressure, and future career choices [33,34,35]. Maladaptive perfectionism and anxiety are often the two most important aspects of mental health problems for university students [36]. According to social cognitive theory, maladaptive perfectionism can be seen as a personality trait, with anxiety viewed as an emotional response and self-compassion as a cognitive process [7,37]. As a personality trait, maladaptive perfectionism affects the individual’s evaluation of setbacks and failures through self-compassion, subsequently affecting their emotional response and psychological state. In the face of difficulties and setbacks, self-compassion can help individuals to understand and accept their situation, thereby reducing negative emotions and stress [38]. Existing research suggests that self-compassion may play a mediating role between maladaptive perfectionism and anxiety in university students [38,39,40]. Maladaptive perfectionists often perceive their own performance too negatively and are unable to accept their failures or imperfections [39]. This attitude makes them more likely to experience anxiety. However, when individuals are able to develop the capacity to be self-compassionate, anxiety symptoms can be relieved through their improved emotional regulation ability [41]. Anxiety is often associated with difficulties in emotional regulation, and self-compassion can help individuals to deal with negative emotions more effectively [38,40]. It provides positive self-talk and alleviates internal self-criticism, thereby helping individuals to better understand and respond to their own emotional states and reduce anxiety [42]. Therefore, this study proposes Hypothesis 2: first-year undergraduate students’ maladaptive perfectionism significantly predicts anxiety through the mediating role of self-compassion.

### 2.3. The Moderating Role of Family Support

Family support, as part of a social support system, is a process in which family members provide each other with emotional, informational, and behavioural support [43]. This support, manifested in various forms such as encouragement, understanding, care, practical advice, and providing solutions, promotes the personal growth of family members, meeting their emotional needs and helping them to cope with difficulties and challenges [44]. Family support can provide emotional support such as understanding, caring, and encouragement [45]. This helps to relieve anxiety and stress and promotes university students’ ability to respond positively to situations with more self-confidence. In addition, family members can provide informational support such as giving practical advice and guidance [46]. Previous studies have found that family support is associated with both maladaptive perfectionism and self-compassion, and that the level of family support may play an important moderating role between maladaptive perfectionism and self-compassion [36,47]. When university students feel a higher level of support from their families, they are more likely to respond positively to challenges and difficulties, reduce the pursuit of maladaptive perfectionism, and focus more on personal growth and progress [48]. Thus, it is crucial to focus on first-year undergraduate students’ emotional needs, provide positive support and encouragement, and help them develop more positive self-evaluation through establishing a good family support system to promote their mental health and growth. Therefore, this study proposes Hypothesis 3: first-year undergraduate students’ maladaptive perfectionism significantly predicts self-compassion through the moderating role of family support.

### 2.4. The Present Study

**Figure 1:**
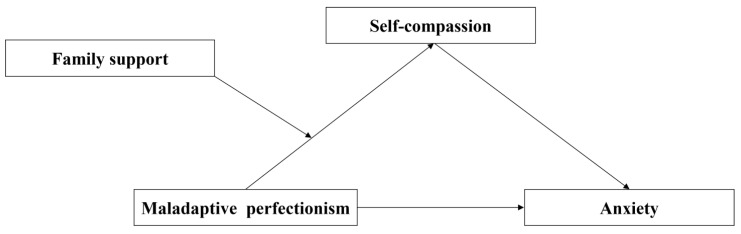
The proposed moderated mediation model.

The present study aims to examine the relationship between Chinese first-year undergraduate students’ maladaptive perfectionism and anxiety. Specifically, we built a moderated mediation model to answer the following questions: (1) Does self-compassion mediate the association between maladaptive perfectionism and anxiety? (2) Does family support moderate the relationship between maladaptive perfectionism and self-compassion (Figure 1)?

## 3. Method

### 3.1. Participants

A convenient random sampling method was adopted. A total of 1000 first-year undergraduate students from four universities in Jiangsu, Zhejiang, and Guangxi were selected as the research subjects. After the removal of 76 invalid questionnaires, such as those with omissions, errors, and irregularity of responses, 924 valid questionnaires (92.40%) were obtained from 170 males and 754 females aged 16~23 years old (M ± SD = 18.60 ± 0.98). The present study was approved by the Research Ethics Committee of Nanjing Normal University of Special Education.

### 3.2. Research Tools

#### 3.2.1. Frost Multidimensional Perfectionism Scale—Brief

This scale adopts the basic version of the perfectionism scale adapted by Burgess [13]. The scale consists of a total of 8 items, comprising the two dimensions of Evaluative Concerns and Striving. The Likert 5-point scoring method was adopted, ranging from 1 (strongly disagree) to 5 (strongly agree). A higher score indicates a stronger tendency towards maladaptive perfectionism. This scale was applied to Asian samples with relatively high reliability and validity [49]. In the present study, the scale’s Cronbach’s α was 0.81, and that of the two fractal dimensions was 0.84 and 0.79.

#### 3.2.2. Anxiety Scale

The Generalized Anxiety Disorder (GAD-7) scale was used to assess the severity of individuals’ generalised anxiety [50]. The questionnaire consisted of 7 items describing typical symptoms of generalised anxiety and asked participants to report how often they had experienced these symptoms over the past two weeks. The 7-point Likert scoring method was adopted, ranging from 0 (not at all) to 3 (almost every day). A higher score indicates a more severe symptom of generalised anxiety. This scale was widely applied to Chinese undergraduate samples with relatively high reliability and validity [51]. In the present study, the scale’s Cronbach’s α was 0.92.

#### 3.2.3. Self-Compassion Scale

The basic version of the self-compassion scale was adopted [52]. The scale consists of a total of 12 items. The 5-point Likert scoring method was adopted, ranging from 1 (strongly disagree) to 5 (strongly agree). A higher score indicates a higher level of self-compassion. In the present study, the scale’s Cronbach’s α was 0.80.

#### 3.2.4. Family Support Questionnaire

The family support dimension of the Multidimensional Perception Social Support Scale was used [53]. The dimension consists of a total of 4 items. The 7-point Likert scoring method was adopted, ranging from 1 (strongly disagree) to 7 (strongly agree). A higher score indicates a higher level of perceived family support. In the present study, the questionnaire’s Cronbach’s α was 0.97.

### 3.3. Statistical Analysis

In the present study, SPSS 26.0 and Process 3.3 were used to conduct statistical data collection and analysis. SPSS 26.0 was used to conduct data entry and collection, descriptive statistical analysis, and related analysis; then, we used Hayes’s Process macro for SPSS and chose Bootstrap to test confidence interval estimation, and the 95% confidence interval (CI) was calculated by repeating the sampling 5000 times [54].

## 4. Results

### 4.1. Common Method Deviation

The present study was based on questionnaires and may have common method biases [55]. Therefore, Harman’s single-factor testing method was used to test for common method biases. The unrotated factor analysis showed that the eigenvalues of six factors were greater than 1. The total variance of the first factor was 27.10%, which was below the critical value of 40%, indicating that there was no serious common method deviation in the present study.

### 4.2. Descriptive Statistics and Related Analysis for Each Variable

Table 1 shows that there was a pairwise correlation between first-year undergraduate students’ maladaptive perfectionism, anxiety, self-compassion, and family support. Maladaptive perfectionism exhibited a significant positive correlation with anxiety and a significant negative correlation with self-compassion and family support; anxiety had a significant negative correlation with self-compassion and family support; and self-compassion exhibited a significant positive correlation with family support. In addition, there were also significant correlations between gender and family support and among Hukou (urban or rural household), anxiety, self-compassion, and family support. Therefore, in the subsequent analysis, these were included as control variables in the model. All variables were normally distributed as the skewness and kurtosis were within range (skewness < |2.0| and kurtosis < |7.0|) [56]. The independence of residuals was satisfied (1 < D-W < 2), and there was no multicollinearity (VIF < 5) [57,58].

### 4.3. Testing for the Mediation Effect

**Figure 2:**
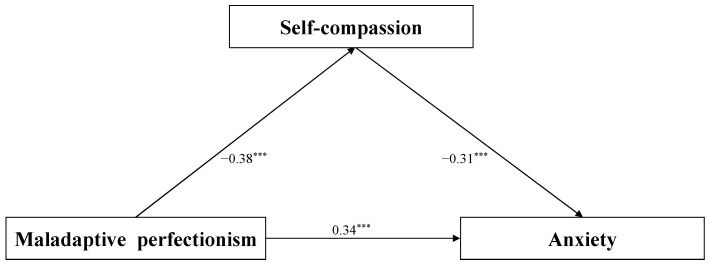
The mediation model. Note: *** *p*  <  0.001.

In order to explore the mediating effect of first-year undergraduate students’ self-compassion between maladaptive perfectionism and anxiety, Hayes’ Model 4 of the PROCESS macro for SPSS was used [54]. The results after controlling for gender and Hukou are shown in Figure 2. The results of the first step indicate that maladaptive perfectionism significantly negatively predicts self-compassion (*β* = −0.38, *p* < 0.001). The results of the second step indicate that maladaptive perfectionism significantly positively predicts anxiety (*β* = 0.46, *p* < 0.001). The results of the third step indicate that self-compassion significantly negatively predicts anxiety (*β* = −0.31, *p* < 0.001), and the positive predicting function of maladaptive perfectionism is still significant in terms of anxiety (*β* = 0.34, *p* < 0.001), which shows that self-compassion plays a mediating role between first-year undergraduate students’ maladaptive perfectionism and anxiety. The bias-corrected percentile bootstrap method was then adopted to examine the indirect effects, with the mediation effect value being 0.12, SE being 0.02, and 95%CI being [0.09, 0.15], accounting for 25% of the total effect. Thus, it can be seen that self-compassion has a significant partial mediating effect between undergraduate maladaptive perfectionism and anxiety.

### 4.4. Test of the Moderated Mediation Model

**Figure 3:**
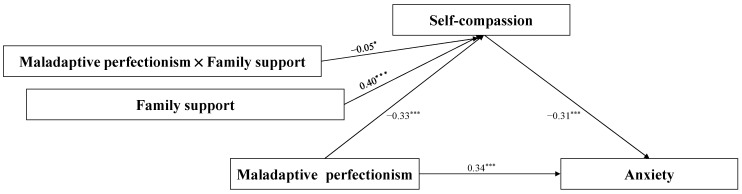
The moderated mediation model. Note: * *p*  <  0.05, *** *p * <  0.001.

In order to explore the moderating role of family support between first-year undergraduate students’ maladaptive perfectionism and self-compassion, Hayes’ Model 7 of the PROCESS macro for SPSS was used [54]. The results after controlling for gender and Hukou are shown in Figure 3. The interaction between family support and maladaptive perfectionism has a significant predictive effect on self-compassion (*β* = −0.05, *p* < 0.05). Thus, it can be seen that family support plays a significant moderating role on the first half of the path of maladaptive perfectionism in the mediation model.

**Figure 4:**
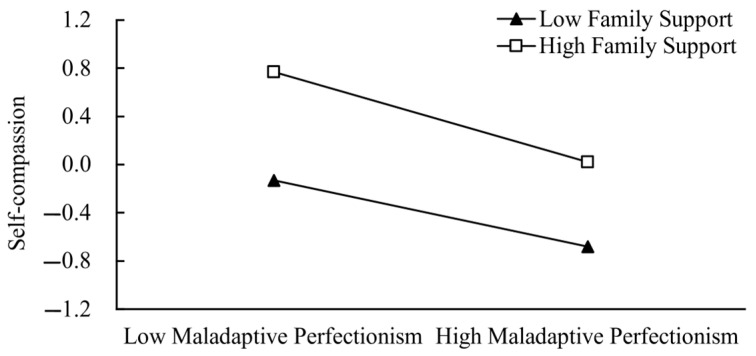
The moderating role of family support.

To further analyse the moderating effect of family support between maladaptive perfectionism and self-compassion, the participants were divided into two groups according to their family support scores: high family support group (*M* + 1*SD*) and low family support group (*M* − 1*SD*). The predictive effects of maladaptive perfectionism on self-compassion were investigated for both groups. As shown in Figure 4, among the first-year undergraduate students with stronger family support, maladaptive perfectionism had a significant negative predictive effect on self-compassion (*β*_simple slope_ = −0.38, *p* < 0.001); for those with less family support, it also had a significant negative predictive effect (*β*_simple slope_ = −0.27, *p* < 0.001), although it was less predictive. Thus, it is found that family support has a significant moderating effect between maladaptive perfectionism and self-compassion.

## 5. Discussion

Emerging adulthood is a transition period in which individuals change from being dependent to independent and become more mature in terms of their psychological and social development. During this transition period, first-year undergraduate students are faced with changes in their living environment, academic expectations, and interpersonal communication, which can lead to various issues that can trigger anxiety and other psychological distress. The present study explored the relationships among undergraduate maladaptive perfectionism, anxiety, self-compassion, and family support and examined the mediating role of self-compassion and the moderating role of family support, providing empirical evidence for undergraduate mental health education. Therefore, for university students, it is recommended that the education and guidance on maladaptive perfectionism be strengthened in order to reduce its negative effect on mental health; to improve individuals’ self-compassion to better cope with the frustration and difficulties in life and relieve negative emotions such as anxiety; and to provide more family support and assistance. These measures will help first-year undergraduate students to better adapt to the transition phase of emerging adulthood and promote healthy growth and development.

### 5.1. The Relationship between Maladaptive Perfectionism and Anxiety

The results show that first-year undergraduate students’ maladaptive perfectionism can significantly positively predict anxiety. The higher the maladaptive perfectionism, the more severe the anxiety, confirming Hypothesis 1. Maladaptive perfectionism refers to a tendency of individuals to strive for perfection and high standards and to demand too much from their own and others’ performance [7]. Anxiety, on the other hand, is a negative emotional state characterised by nervousness, worry, and unease [59]. Studies have found a close relationship between maladaptive perfectionism and anxiety [60]. Within a diathesis–stress model, perfectionism is considered a potential vulnerability [61]. When individuals face challenges or adversities, this tendency towards maladaptive perfectionism may increase their risk of experiencing anxiety. Maladaptive perfectionists often set excessively high standards and goals for themselves, striving for flawless performance and achievement [7]. However, it is difficult to achieve such idealised standards in real life, so they often feel disappointed and frustrated and may blame themselves [62]. This constant pursuit of perfection increases the likelihood of anxiety when faced with failure and setbacks [25]. In addition, compared to middle and high school students, first-year undergraduate students are at a more mature stage of cognitive development. They are more motivated to pursue success and tend to closely associate self-worth with achievement [63]. They believe that only by performing perfectly can they gain recognition and affirmation from others, but this exacerbates feelings of anxiety. Therefore, parents and teachers should guide maladaptive perfectionists to focus more on the positive aspects of life, reduce excessive responses to anxiety, and avoid cognitive and behavioural dysfunction resulting from setbacks in self-esteem and confidence. When guiding first-year students to set personal goals, they should be encouraged to pursue challenging goals rather than overly high and unrealistic ones. Particularly when faced with mistakes and failures, efforts should be made to increase their ability to cope with setbacks with more courage.

### 5.2. The Mediating Role of Self-Compassion

The present study found that self-compassion plays a partial mediating role between maladaptive perfectionism and anxiety; that is, maladaptive perfectionism in undergraduate students affects anxiety through the mediating role of self-compassion, verifying Hypothesis 2. Self-compassion can play a protective role in inhibiting the development of anxiety and improving anxiety symptoms, which is especially important in terms of undergraduate mental health [64,65]. In real life, university students are often faced with various challenges and difficult situations not long after enrolment, including problems such as academic pressure, social difficulties, and future career planning [33,34,35]. Maladaptive perfectionists tend to feel frustrated and depressed because they cannot meet idealised standards, which further increases their anxiety. However, due to the influence of traditional Chinese culture and the stigmatisation of mental illness, university students often find it difficult to share their psychological and spiritual feelings with strangers such as psychologists and psychological consultants [66,67]. Self-compassion is a mild approach to emotional regulation that does not require university students to see themselves as better or perfect persons [68]. Instead, this approach may help university students cope with their own deficiencies or difficulties in a healthier way, thereby relieving the negative impact of maladaptive perfectionism and reducing negative emotions [69]. Through self-compassion, university students can deal with their own deficiencies and difficulties in a milder way, understand their own feelings and emotions, and give themselves more care and support. This approach can help them to build positive self-images and reduce the emotions of self-blaming and self-denying, thus reducing anxiety [70]. Therefore, self-compassion, as a positive emotional regulation strategy, can help first-year undergraduate students to develop healthier coping strategies and is also an important factor in reducing anxiety. As adolescents transition to college life, a crucial period of physical and psychological development, school mental health education should focus on exploring the positive psychological qualities of college students, with an emphasis on cultivating self-compassion. Regularly conducting mental health lectures or group counselling activities that focus on self-compassion can guide college students to approach potential setbacks and difficulties with a more tolerant and benevolent attitude, thereby preventing and alleviating feelings of anxiety.

### 5.3. The Moderating Role of Family Support

The present study found that family support plays a moderating role between maladaptive perfectionism and self-compassion, with maladaptive perfectionism having less of an impact on self-compassion among first-year undergraduate students with strong family support than among those with less support, verifying Hypothesis 3. The results of this study are consistent with the risk-buffering model, which emphasises that family support can mitigate the adverse effects of risk factors and that individuals with positive external resources are more resilient in the face of external challenges [71]. A possible explanation is that the family is an important living environment for university students in the process of growing up, as well as being an important social support system [43]. Compared with that of other university students, the mental health of first-year undergraduate students is much more easily affected by the family environment. These students are faced with many challenges and pressures, including the influence of maladaptive perfectionism. Family support plays a very important role in university life. Higher levels of family support can help first-year students to develop more positive self-perception and a realistic and healthy understanding of their abilities and shortcomings [72]. This positive self-cognition can lead to a better understanding of their own advantages and disadvantages, as well as acceptance of their own imperfections [48]. In addition, higher levels of family support can promote self-compassion in first-year undergraduate students. When faced by setbacks and failures, having family support and understanding can make it easier for these students to accept their own shortcomings and learn from them [73]. The present study found that strong family support moderated maladaptive perfectionism. However, first-year undergraduate students who lacked family support struggled to achieve this positive effect and may not receive the necessary understanding and comfort from family members, leading to difficulties in accepting their inadequacies and learning from their failures [74]. Therefore, through encouragement and praise, family members can boost the self-confidence of first-year students so that they become more proactive, motivated, and confident in pursuing their goals during university life. At the same time, parents should avoid setting unreasonable and unrealistic academic standards when expressing their expectations of their children and, instead, show more concern. When faced with failures or setbacks, family members can offer understanding and comfort, helping them to understand that failure is part of growth and not a measure of personal worth. This not only satisfies the individual’s need for belonging and respect but also facilitates the development of the individual’s self-potential. Ultimately, it reduces the negative effects of maladaptive perfectionism and improves the mental health of first-year undergraduate students.

### 5.4. Limitations

The present study has several limitations that should be considered. First, although there is some theoretical basis to the cross-sectional study, no causal inference can be made. Future studies could use longitudinal methods or intervention experiments to examine the moderated mediation model in this study and explore the causal relationship between maladaptive perfectionism and anxiety. At the same time, we can further explore the mediating role of other psychological resource variables (such as psychological flourishing and self-efficacy), determine key psychological resource variables, and integrate these into a comprehensive high-order concept to verify the mediating role of all psychological resources. In addition, by recruiting students from different grades to participate, the external validity of the study can be improved, and more information and evidence can be obtained for the development of targeted intervention programmes.

## 6. Conclusions

Maladaptive perfectionism has a significant positive predictive effect on anxiety. Moreover, it can not only directly predict anxiety, but can also indirectly affect anxiety through self-compassion, which plays a partial mediating role between maladaptive perfectionism and anxiety. Family support plays a moderating role between maladaptive perfectionism and self-compassion. When the level of family support is relatively high, the negative predictive effect of maladaptive perfectionism on self-compassion is significantly weakened.

## Figures and Tables

**Table 1:** Descriptive statistics and correlations among variables (N = 924).

Variable	*M*	*SD*	1	2	3	4	5	6
1. Gender	1.82	0.39	1					
2. Hukou	1.45	0.50	0.08 *	1				
3. Maladaptive perfectionism	20.85	5.40	−0.04	−0.02	1			
4. Anxiety	11.75	4.46	−0.02	−0.08 *	0.46 **	1		
5. Self-compassion	41.00	6.76	0.02	0.07 *	−0.38 **	−0.44 **	1	
6. Family support	20.76	4.89	0.09	0.07 *	−0.12 **	−0.17 **	0.45 **	1

Note: gender: 1 = female, 2 = male; Hukou: 1 = urban, 2 = rural; *M* is mean value; *SD* is standard deviation. * *p*  <  0.05, ** *p*  <  0.01.

## Data Availability

The data presented in this study are available on request from the corresponding author.
